# Effects of open‐label sesame oil applied to cardiac surgery patients in preventing amiodarone‐induced phlebitis: A randomized controlled trial

**DOI:** 10.1111/nicc.13085

**Published:** 2024-04-27

**Authors:** Muaz Gülşen, Sevban Arslan

**Affiliations:** ^1^ Surgical Nursing Department, Faculty of Health Sciences Çukurova University Adana Turkey

**Keywords:** amiodarone, nursing care, phlebitis, sesame oil, traditional medicine

## Abstract

**Background:**

Amiodarone is a prophylactic rhythm‐regulating drug used to prevent arrhythmia; However, especially during infusion, it has the potential to cause a number of complications, especially phlebitis.

**Aim:**

The aim of the study is to determine the effects of sesame oil, which has the potential to prevent phlebitis that may occur during amiodarone infusion administered to patients after cardiac surgery.

**Design:**

This prospective, two‐arm (1:1), block randomized controlled interventional study.

**Methods:**

This study was conducted with 44 patients treated in the coronary intensive care unit of a university hospital, who received parenteral infusion of amiodarone. Sesame oil was applied superficially by applying 10 drops to a 10 cm perimeter of the cannula for 10 min. This application was repeated every 8 h during the 24‐h amiodarone infusion. No intervention was made to the patients in the control group. However, standard nursing care measures and a standard transparent dressing were applied to the patients in both groups during the peripheral catheter application phase. Patients in the intervention and control groups were evaluated in terms of phlebitis at the end of every 24 h using the Visual Infusion Phlebitis Scale. The study was reported according to the CONSORT declaration.

**Results:**

Phlebitis symptoms occurred in 15/22 (68.2%) of the patients in the intervention group on the first day, 3/22 (13.6%) on the second day and 2/22 (9.1%) of the patients on the third day, while in the control group, 20/22 (90.9%) of the patients had phlebitis on the first day and 2/22 (9.1%) on the second day. The incidence of phlebitis was 20/22 (90.9%) in the intervention group and 22/22 (100%) in the control group. There was no statistically significant difference in phlebitis symptoms between groups.

**Conclusion:**

The research results showed that the application of sesame oil did not significantly reduce the frequency of phlebitis. However, a trend indicating delayed onset of phlebitis symptoms was observed in the sesame oil group. Nevertheless, larger sample studies are needed. These studies are expected to assist in determining the effects of sesame oil on phlebitis more precisely and provide stronger support for the results.

**Relevance to Clinical Practice:**

Training of nurses on non‐pharmacological methods should be supported and opportunities should be given for their application.

**What is known about the topic**

- Amiodarone infusion can also be used as a prophylactic rhythm regulator to prevent arythmia in patients who have underwent cardiac surgery.
- However, this can also result in many complications during the infusion, the most common being phlebitis.
- There are studies in the literature showing that pharmacological and phytotherapeutic products are used together to protect patients from this complication.
- Sesame oil is a topical intervention method used to prevent phlebitis with its antioxidant, anti‐inflammatory and antibacterial effects.

**What this paper adds**

- The topical application of sesame oil during amiodarone infusion was found not to have reduced the incidence of phlebitis in patients undergoing cardiac surgery.
- Although not statistically significant, symptoms of phlebitis were observed to appear later, specifically on the 3rd day, in patients treated with sesame oil.

## 1 INTRODUCTION

Today, health issues, such as supraventricular tachycardia, atrial fibrillation and ventricular arrhythmia, threaten the lives of individuals. One of the methods used in the treatment of these adverse conditions is amiodarone infusion.^1^, ^2^ Amiodarone can also be used as a prophylactic rhythm regulator to prevent arythmia in patients who have underwent cardiac surgery.^3^ However, this can also result in many complications during the infusion, the most common being phlebitis.^2^, ^4^, ^5^, ^6^, ^7^ Some further studies suggest that the incidence of phlebitis because of amiodarone infusion varies between 25% and 67%.^5^, ^6^, ^7^, ^8^, ^9^ As shown in above studies, there are disagreements in the scientific literature regarding the clinical incidence.^10^, ^11^, ^12^ In a recent meta‐analysis, total of 35 studies and 15 791 patients examining the frequency and severity of phlebitis were evaluated. As a result of the meta‐analysis, it was stated that the incidence of phlebitis was 31% and 4% of the developing phlebitis cases were serious enough to require intervention.^13^ However, while the Infusion Nurses Association (INS) emphasizes that phlebitis is a preventable complication, it states that the acceptable phlebitis rate in patients treated with amiodarone infusion should be less than 5%.^14^

Under normal conditions, the use of a central venous catheter is preferred during amiodarone infusion to prevent phlebitis.^15^, ^16^ However, in emergencies or short‐term infusions, the peripheral intravenous catheter, which causes less pain, infection and trauma, is preferred.^7^, ^15^ When the peripheral intravenous catheter is preferred, methods, such as dilution of drugs,^17^ heparin^17^ or topical corticosteroids,^18^ are used to prevent the development of phlebitis. During these applications, bleeding, thrombocytopenia, pain and signs of infection because of impaired defence system are detected in patients.^3^, ^4^

There are studies in the literature showing that pharmacological (anticoagulants, anti‐inflammatory and vasodilators) and phytotherapeutic products (chamomilla recutita, notoginseny, aloe vera and sesame oil) are used together to protect patients from this complication.^10^, ^12^, ^19^, ^20^, ^21^ Sesame oil, which is among the phytotherapeutic products, is preferred because it is simple to use, economical and accessible.^3^, ^10^, ^15^, ^22^ Additionally, sesame oil is a topical intervention method used to prevent phlebitis with its antioxidant, anti‐inflammatory and antibacterial effects.^3^, ^23^, ^24^, ^25^

There are studies in the literature on the effectiveness of chamomile oil in preventing phlebitis caused by amiodarone infusion^15^ and of sesame oil in preventing phlebitis caused by chemotherapy drugs.^23^ However, studies evaluating the effectiveness of sesame oil in preventing phlebitis because of amiodarone infusion are limited.^3^ The study was planned inspired by the study conducted by Bagheri‐Nesami et al.^3^ In our study, the sample size, the amount of sesame oil used and the duration were changed. These differences may increase or decrease the effect of sesame oil on phlebitis symptoms.

## 2 AIM OF THE STUDY

The aim of the study was to determine the effects of sesame oil, which has the potential to prevent phlebitis that may occur during amiodarone infusion administered to patients after cardiac surgery.

### 2.1 Research hypotheses

There is no difference between the groups in terms of the effect of sesame oil in preventing phlebitis, which may occur during IV infusion of amiodarone in patients undergoing cardiac surgery.

There is a difference between the groups in terms of the effect of sesame oil in preventing phlebitis, which may occur during IV infusion of amiodarone in patients undergoing cardiac surgery.

## 3 MATERIALS AND METHODS

### 3.1 Type of study

**FIGURE 1:**
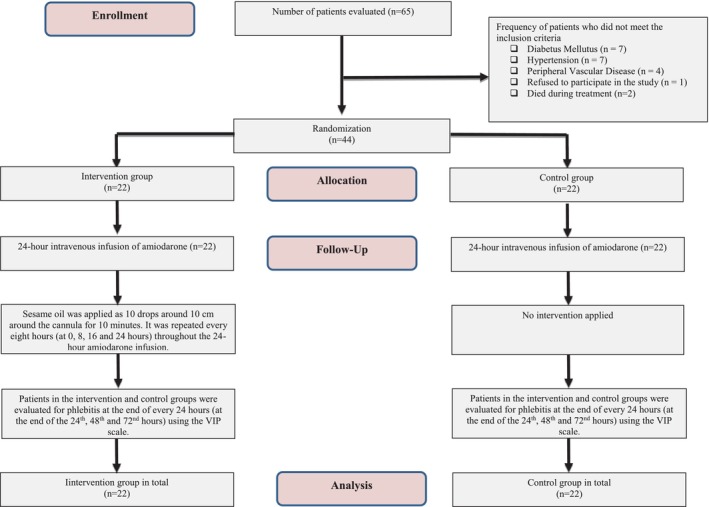
CONSORT flow diagram.

The research was conducted as an open‐label prospective, two‐arm (1:1) block‐randomized controlled study in the coronary intensive care unit (CICU) of a university hospital. The study was reported using the CONSORT protocol (Figure 1 and See File S1). A clinical trial registration number was obtained prior to implementing the study.

### 3.2 Sample of the study

The data were collected between 15 August and 15 October 2023. The population of the study consisted of patients hospitalized in CCUs after cardiac surgery between these dates and treated with IV amiodarone infusion. The sample size was calculated based on the study results of Bagheri‐Nesami and associates.^3^ As a result of this study, the development of phlebitis was found to be 38% in the group using sesame oil, while it was 77% in the control group. According to the Pocock Sample Size calculation formula, the sample size was calculated to have a 95% confidence interval and a power of 0.80,^26^ and the required number of patients in both groups was determined as at least 22.

### 3.3 Inclusion and exclusion criteria

Patients who had previously undergone heart surgery, were over 18 years of age, were conscious, able to communicate, had no sesame allergy, were administered prophylactic heart rhythm regulator amiodarone as treatment, had a stable hemodynamic status and had a peripheral venous catheter, were included in the study. In addition, patients with symptoms of phlebitis for the first time were included in the study to objectively evaluate the symptoms (especially pain) seen in the stages of phlebitis, understand the first signs of the disease and determine how effective sesame oil intervention is in its early stages. Otherwise, the experience of phlebitis could affect individuals' perception and reactions to phlebitis, and this effect could cause symptoms to be hidden.

However, individuals with cancer, diabetes, hypertension and peripheral vascular disease were excluded from the study because of the increased risk of phlebitis.^3^ Additionally, patients who had a myocardial infarction during the procedure received immunosuppressive therapy, underwent resuscitation or could not use an IV catheter for any reason were excluded from the study.

### 3.4 Randomization and assignment to groups

Block randomization was performed in the study to eliminate selection bias and ensure balance in the number of patients between groups. Six blocks of four consisting of the letters A and B were created for the random assignment of patients who met the inclusion criteria, signed the informed consent form and filled in the pretest data. Then, for randomization, a random number list was created in the computer environment, using www.randomizer.org, which assigned individuals to two equal groups: intervention and control groups. Blocks were sorted according to this created number list. These random numbers obtained were reported to the practitioner by an independent researcher (3rd person) who was not involved in the research, before assignment to the two groups, respectively. The monitoring and follow‐up of the randomization process were carried out by an independent researcher who was not involved in the study by creating a follow‐up file. Thus, selection bias was controlled by hiding the randomization and random assignment process in the study.

### 3.5 Blinding

Since sesame oil is an essential oil, it emits an aromatic scent to the environment it is in. For this reason, the patients and the researcher who performed the intervention could not be blinded in terms of the materials used. However, the research data were coded into the SPSS program by an independent researcher, using the letter ‘A’ for the intervention group and the letter ‘B’ for the control group. In this way, it is aimed to blind the statistician in terms of research groups. After the research report was written, the letter ‘A’ was changed to the intervention group and the letter ‘B’ was changed to the control group by the researcher.

### 3.6 Data collection tools

#### 3.6.1 Personal information form

The variables included in the personal information form were selected in accordance with the purpose, hypothesis and target audience of the research. In addition, these selected variables (age, gender, body mass index [BMI], fasting blood sugar level, peripheral catheter site) were determined by citing similar studies in the literature. In this way, it was aimed to increase the reliability and validity of the research.^3^, ^15^, ^24^

#### 3.6.2 Visual infusion phlebitis scale (VIP)

The scale, which was issued by the Infusion Nurses Society, consists of four stages.^14^, ^27^ The scale investigates the potential risks of the catheter when administering peripheral venous therapy and rates the developmental stages of phlebitis. Scores range from zero to four. The validity and reliability of the VIP scale were confirmed, and the interobserver reliability was found with a correlation coefficient of ≥0.85^14^, ^27^ (Table 1).

**TABLE 1:** Visual infusion phlebitis scale.

Staging	Clinical symptoms and findings
Stage 0 (No phlebitis symptoms)	No pain, redness/oedema at the IV site
Stage I (Early symptoms of phlebitis)	In the IV intervention site Redness and/or pain
Stage II (Medium stage of phlebitis)	In the IV intervention site Redness Pain and/or oedema
Stage III (Advanced stage)	In the IV intervention zone Redness Pain and/or oedema Vein palpation
Stage IV (Advanced stage of thrombophlebitis)	In the IV intervention site Redness Pain and/or oedema Vein palpation High fever

### 3.7 Execution

The American Association of Infusion Nurses recommends that the forearm be preferred for amiodarone infusion, despite the low level of evidence (level IV).^14^ This recommendation also coincides with the fact that the standard application area for peripheral intravenous catheter placement in the intensive care unit where the research was conducted was the forearm. The proximal region of the upper extremity (forearm) was preferred as the application area in the study because of its ease of observation and application.

In order to prevent any inconsistency in peripheral intravenous catheter application and increase reliability and validity, the nurse in charge of the intensive care unit applied peripheral intravenous catheter to both patient groups with a 20‐gauge cannula. During the preparation phase of peripheral intravenous catheter application, the nurse in charge washed her hands and took care to use gloves and masks. In order to reduce the risk of infection during the procedure, a sterile working environment was created and a skin antiseptic solution determined by the hospital management (content: 0.1% Octenidine Hydrochloride, 2% Phenoxyethanol) was used to disinfect the selected vein. In order to minimize the risk of phlebitis that could occur because of peripheral intravenous catheterization attempts other than amiodarone infusion, standard nursing care measures were applied to the patients in both the application and control groups during the peripheral catheter application phase. The peripheral intravenous catheter was secured with standard clear dressing (Tegaderm).

Amiodarone infusion was administered through the inserted peripheral intravenous catheter for only 24 h (300 mg amiodarone in 150 cc 5% Dextrose in 10 min +900 mg amiodarone in 500 cc 5% Dextrose, 33 mL/h in the first 6 h, 17 mL/h in the next 18 h). The use of in‐line filters was not implemented during amiodarone administration. There is no clear procedure in this regard in the institution and the intensive care unit.

Patients in both groups were informed about the purpose of the study and the procedures to be performed. Informed patients were made to sign an ‘Informed Consent Form’. After obtaining the necessary permissions, the ‘Personal Information Form’ was filled out using the face‐to‐face interview method. Then, the third person who performed the randomization process was called by phone to find out which group the patient was assigned to. The process up to this point was implemented homogeneously between the intervention and control groups.

Topical sesame oil was applied to patients in the intervention group. The applied sesame oil is not subject to prescription in the country where the research was conducted. Sesame oil (Talya Oleum Sesamum Indicum L. Sesame Oil 20 mL) is approved by the Ministry of Agriculture and Forestry (Business Registration Certificate No: TR‐K‐003391) and is produced in accordance with the Food Codex. Topical sesame oil was applied superficially, at equal intervals, 1 drop per minute, 10 cm around the peripheral intravenous catheter, and this process took 10 min in total. At the end of the period, the peripheral intravenous catheter area was covered with standard transparent dressing (Tegaderm). This application was repeated every 8 h (at 0, 8, 16 and 24 h) throughout the 24‐h amiodarone infusion.

During the application, sesame oil was applied superficially to the tissues without manipulation, in order to prevent the redness, which may occur on the skin surface because of the vasodilator effect of the massage from being confused with the symptoms of phlebitis. Additionally, in order to avoid any doubt as to whether massage or sesame oil prevents the formation of phlebitis, sesame oil application was carried out superficially, not together with massage.

No intervention was made to the patients in the control group. In the patients in the control group, the peripheral intravenous catheter was covered with transparent dressing material (Tegaderm) in order to evaluate its surroundings for phlebitis.

After all applications, patients in the intervention and control groups were evaluated for phlebitis using the VIP scale at the end of every 24 h (at the end of the 24th, 48th and 72nd hours). The highest of the phlebitis stages obtained by the patients as a result of the evaluation was accepted as the final evaluation (Figure 1).

When phlebitis was detected in both groups, the peripheral intravenous catheter was removed and standard care of the intensive care unit (cold compress to the area) was applied. If necessary, painkillers and antibiotics were administered according to the doctor's instructions. However, no painkillers or antibiotic treatments were needed during the study.

### 3.8 Assessment of the study

Coding and evaluation of the data were done in computer setting using SPSS 22.0 (International Business Machines‐IBM, New York, USA) program. The results were evaluated at 95% confidence interval and 0.05 statistical significance level. Descriptive statistics, such as frequency, mean and standard deviation, were used in evaluating the data. In inferential analysis, chi‐square, independent t‐test and Fisher's exact test were used.

### 3.9 Ethical considerations of the study

Before collecting the research data, Cukurova University Non‐Interventional Clinical Research Ethics Committee Permission (Date: 03/08/2023, Number of Meetings/Decision no: 142/14) was obtained from the Chief Physician. In addition, the patients who accepted to participate in the study were informed about the purpose of the study and the procedures to be performed and they signed the ‘Informed Consent Form’.

## 4 RESULTS

### 4.1 Findings regarding the descriptive characteristics of the patients

The mean age of the patients in the intervention group was 56.70 ± 7.06, and 63.6% of the patients were male. The mean age of the patients in the control group was 56.04 ± 7.31, and 40.9% of the patients were male. The patients in the intervention and control groups were similar in terms of descriptive characteristics (*p* values are .131, .983, .065 and .937, respectively; Table 2).

**TABLE 2:** Findings regarding descriptive characteristics and the comparison of the patients.

	Intervention group	Control group	*p* Value
Gender (*n* (%))			
Male	14 (%63.6)	9 (%40.9)	.131^a^
Female	8 (%36.4)	13 (%59.1)	
Age (mean + standard deviation, year)	56.70 ± 7.06	56.04 ± 7.31	.983^b^
Body Mass Index (kg/m^2^)	25.80 ± 1.39	26.66 ± 1.58	.065^b^
Fasting Blood Sugar (mg/dL)	94.95 ± 7.95	94.77 ± 7.18	.937^b^

^a^ Pearson Chi‐square test.

^b^ Student's *t*‐test.

### 4.2 Findings regarding comparison of the frequency of 3‐day phlebitis of patients

The comparison of the incidence of phlebitis between the intervention and control groups is presented in Table 3. In the study, 68.2% (15/22) of the patients in the intervention group had phlebitis symptoms on the first day, 13.6% (3/22) on the second day and 9.1% (2/22) on the third day. 90.9% (20/22) of the patients in the control group had phlebitis symptoms on the first day and 9.1% (2/22) on the second day. While the incidence of phlebitis was 90.9% (20/22) in the intervention group, it was 100% (22/22) in the control group, and there was no statistically significant difference between the groups (*p* = .091; Table 3).

**TABLE 3:** Comparison of the frequency of 3‐day phlebitis of patients.

Groups	*n*	Incidence of phlebitis (*n* (%))			
		First Day^c^ (At the end of the 24th hour)	Second day^c^ (At the end of the 48th hour)	Third day^c^ (At the end of the 72th hour)	Total
Intervention	22	15 (%68.2)	3 (%13.6)	2 (%9.1)	20 (%90.9)
Control	22	20 (%90.9)	2 (%9.1)	0 (%0.0)	22 (%100)
*p* Value		.132^a^	1.00^a^	.351^a^	.091^b^

^a^ Fisher's exact test.

^b^ Likelihood ratio.

^c^ Incidence of phlebitis only on the specified day.

### 4.3 Findings regarding the comparison of patients' clinical symptoms and phlebitis level

Table 4 presents the comparison of the patients' clinical findings and phlebitis levels. The numbers and percentages of patients in different stages, namely the first day, second day and third day, are given for the intervention and control groups.

**TABLE 4:** Comparison of patients' clinical signs/signs and phlebitis level.

	Intervention group (*n*/*N* (%))				Control group (*n*/*N* (%))				*p* Value
	First Day	Second Day	Third Day	Total	First Day	Second Day	Third Day	Total	
Stage 0 (No phlebitis symptoms)									
No pain, redness/oedema at the IV site	–	–	–		–	–	–		
Stage I (Early symptoms of phlebitis)									
In the IV intervention site redness	14/22 (%63.6)	1/22 (%4.5)	–	15/22 (%68.1)	11/22 (%50)	–	–	11/22 (%50)	.220^a^
In the IV intervention site pain	14/22 (%63.6)	1/22 (%4.5)	–		11/22 (%50)	–	–		
Stage II (Medium stage of phlebitis)									
In the IV intervention site redness	1/22 (%4.5)	2/22 (%9.1)	–	3/22 (%13.6)	6/22 (%27.3)	–	–	6/22 (%27.3)	.262^a^
In the IV intervention site pain	1/22 (%4.5)	2/22 (%9.1)	–		6/22 (%27.3)	–	–		
In the IV intervention site oedema	1/22 (%4.5)	2/22 (%9.1)	–		6/22 (%27.3)	–	–		
Stage III (Advanced stage)									
In the IV intervention zone redness	–	–	2/22 (%9.1)	2/22 (%9.1)	3/22 (%13.6)	2/22 (%9.1)	–	5/22 (%22.7)	.216^a^
In the IV intervention site pain	–	–	2/22 (%9.1)		3/22 (%13.6)	2/22 (%9.1)	–		
In the IV intervention zone oedema	–	–	2/22 (%9.1)		3/22 (%13.6)	2/22 (%9.1)	–		
In the IV intervention zone vein palpation	–	–	2/22 (%9.1)		3/22 (%13.6)	2/22 (%9.1)	–		
Stage IV (Advanced stage of thrombophlebitis)									
In the IV intervention zone redness	–	–	–		–	–	–		
In the IV intervention site pain	–	–	–		–	–	–		
In the IV intervention zone oedema	–	–	–		–	–	–		
In the IV intervention zone vein palpation	–	–	–		–	–	–		
In the IV intervention zone high fever	–	–	–		–	–	–		
Total	15/22 (%68.1)	3/22 (%13.6)	2/22 (%9.1)	20/22 (%90.8)	20/22 (%90.9)	2/22 (%9.1)		22/22 (%100)	.091^b^

Abbreviation: IV, Intravenous.

^a^ Pearson chi‐square test.

^b^ Likelihood ratio.

On the first day, redness and pain (Stage I) were observed in 14 of 22 patients in the intervention group, and redness, pain and edema (Stage II) were observed in 1 patient at the peripheral venous catheter entry site. In the control group, 11 out of 22 patients had redness and pain (Stage I), 6 of them had redness, pain and edema (Stage II) and 3 of them had redness, pain, edema and venous cord stiffness (Stage III).

On the second day, redness and pain (Stage I) were observed in 1 of 22 patients and redness, pain and edema (Stage II) were observed in 2 patients at the peripheral venous catheter entry site in the intervention group, while redness, pain, edema and venous cord were observed in 2 of 22 patients in the control group and hardness (Stage III) was detected.

On the third day, redness, pain, edema and venous cord stiffness (Stage III) were detected in 2 of 22 patients at the peripheral venous catheter entry site in the intervention group.

When comparing the groups in terms of phlebitis stages, it was found that there was no statistically significant difference (*p* = .091). However, in the intervention group (*n*/*N* = 20/22), a lower number of phlebitis cases were observed, with findings appearing on the first day in 68.1% of the patients. In contrast, the control group exhibited a higher frequency of phlebitis on the first day, at 90.9% (Table 4).

In the study, when the phlebitis symptoms (redness, pain, swelling, stiffness, palpable venous cord and purulent discharge) experienced by the patients in the intervention and control groups were compared on different days, it was determined that there was no statistically significant difference between the groups. However, it was found that fewer symptoms occurred in the intervention group these days (Table 5).

**TABLE 5:** Comparison of the patients' phlebitis symptoms.

Symptoms	Intervention group (*n*/*N* (%))	Control group (*n*/*N* (%))	*p* value
Redness	20/22 (%90.9)	22/22 (%100)	.488^a^
Pain	20/22 (%90.9)	22/22 (%100)	.488^a^
Swelling/Oedema	5/22 (%22.7)	11/22 (%50)	.060^b^
Palpable Venous Cord	2/22 (%9.1)	5/22 (%22.7)	.412^a^
Purulent Drainage	0/22 (%0.0)	0/22 (%0.0)	–

*Note*: Data are stated as frequency and percentages on different days.

^a^ Fisher's exact test.

^b^ Pearson chi‐square test.

## 5 DISCUSSION

In cardiac surgery, which is a traumatic surgical intervention that weakens the immune system of the patient, a central venous catheter is applied to patients. It is known that this intervention may damage the patient's skin barrier, allowing microorganisms to enter through the PIVC, and even cause phlebitis. Control and prevention of phlebitis development in patients receiving amiodarone infusion after cardiac surgery are important nursing interventions.^5^, ^10^, ^28^

When comparing the findings related to demographic characteristics of the groups, the mean age of the patients in both groups was similar. Also, in other studies in the literature, the average age of intervention and control groups was similar.^3^, ^9^, ^29^ Similarity in findings might be a result of selecting the patients from the CICU and because cardiovascular diseases occur at advanced ages.^3^ In the study of Mandal and associates, the risk of developing phlebitis was higher in patients over the age of 60.^29^ In this study, the mean age of the patients was close to 60 years.

There was no statistically significant difference between the groups in terms of gender (Table 2). While there are studies in the literature indicating that gender does not affect the incidence of phlebitis,^3^ there are also studies both suggesting that the risk of developing phlebitis is higher in males^30^ as well as that it is higher in females.^29^, ^31^ Given such divergent results, conducting further epidemiological studies is recommended to examine the relationship between gender and the incidence of phlebitis.

In this study, no statistically significant difference was found in terms of the incidence of phlebitis in patients in the intervention and control groups. However, it was determined that sesame oil applied topically during infusion to cardiac surgery patients reduced the incidence of phlebitis by 10% compared with the control group. In addition, phlebitis symptoms appeared later (on the 3rd day) in patients who received sesame oil intervention (Table 3). Studies in the literature with patients receiving chemotherapy,^23^ and patients treated in the CICU,^3^, ^15^ support these results. In the study of Han and associates (2022), where transparent dressing (group A) and sterile gauze (group B) were compared, the incidence of 2nd degree phlebitis in group A was significantly less than in group B after 10 days of follow‐up.^5^ The high incidence of phlebitis in patients receiving standard care shows that it is important to apply different nursing interventions. In this study, the effect of locally applied sesame oil on reducing the incidence of phlebitis may have been caused by its antioxidant, anti‐inflammatory and antibacterial qualities.^3^, ^23^, ^24^, ^25^, ^32^

Despite the positive effects of the sesame oil application in the intervention group, there was no significant difference in the incidence of phlebitis between the intervention and the control groups (Table 3). These results might be because of the limitations, such as the short duration of the study and selecting patients from a single clinic. To determine the efficacy of sesame oil, carrying out simultaneous and longer studies in different clinics is recommended for future studies.

It was determined that there was no statistically significant difference when comparing the findings regarding phlebitis stages between the groups. However, it was determined that the number of patients with stage 2 and Stage 3 phlebitis in the intervention group was lower than in the control group (Table 4). It was found that phlebitis symptoms occurring in the second and third stages were less common in patients in the intervention group (Table 5). Similar studies in the literature support the finding that sesame oil application reduces phlebitis symptoms.^3^, ^5^, ^22^, ^23^ Sesame oil prevents the formation of inflammatory response and prevents the formation of phlebitis, thanks to its antioxidant lignans (sesamin and sesaminol), and anti‐inflammatory agents, such as interleukin and endothelin.^32^ There are also studies with similar results in the literature.^22^

In a study conducted with patients undergoing cardiac surgery, it was stated that sesame oil reduced the symptoms of phlebitis and therefore prevented the formation of advanced stages.^5^ There are studies in the literature that support the conclusion that the application of sesame oil reduces the symptoms of phlebitis.^3^, ^22^, ^23^ Sesame oil prevents the inflammatory response and phlebitis formation thanks to the antioxidant lignans (sesamin and sesaminol) it contains, agents such as interleukin and endothelin‐1.^22^, ^32^ In a meta‐analysis study, the effects of substances such as ichthammol glycerin, heparinoids, magnesium sulfate glycerin, aloe vera, sesame oil, massage, alcohol and hot and cold applications on phlebitis symptoms were investigated. It was revealed that sesame oil reduces the frequency of all symptoms related to phlebitis, especially pain.^10^ In another study conducted in line with these results, it was reported that sesame oil was effective in both the prevention and treatment of phlebitis.^10^, ^25^ In another study, palpable venous cord symptoms were observed in some patients at the end of the 24th and 48th hours in the group treated with nitroglycerin ointment, while no such symptoms were observed in the 72‐h period in the group treated with sesame oil.^33^

### 5.1 Limitations

For the sample selection of the study, patients treated in a single intensive care unit were included. In addition, only 20‐G catheters are used in the intensive care unit where the research was conducted. Therefore, no judgement could be made about the effect of the application on the use of catheters of different sizes. Limitations include the inability to administer a placebo to the control group because of the aromatic smell of sesame oil, the small sample size and the inability to blind the patients and the researcher.

### 5.2 Clinical implications

As a result of the research, no negative effects of sesame oil were observed in patients undergoing cardiac surgery. The results obtained are in parallel with similar studies.^3^, ^10^, ^23^, ^33^ Considering the results in the literature, it can be said that topically applied sesame oil is a simple, effective and safe method to use in the clinic to prevent phlebitis, which may occur because of amiodarone infusion in patients undergoing cardiac surgery. Therefore, it is a suitable approach to the management of phlebitis symptoms within the scope of complementary medicine. However, because of the high incidence of phlebitis and the existence of many different intravenous treatment contents, clinical practice guidelines such as the Joanna Briggs Institute (JBI) and the Registered Nurses Association of Ontario (RNAO) emphasize that this issue needs to be further investigated.

## 6 CONCLUSIONS

The study revealed that application of sesame oil did not statistically significantly reduce the frequency and symptoms of phlebitis. However, it was observed that the appearance of phlebitis symptoms was delayed in the sesame oil group. In future studies, it is recommended to take the odour of sesame oil into consideration and seek advice from a pharmacologist and make it odourless. In studies with odourless sesame oil, it is important to blind patients and researchers and use the placebo method. Additionally, a similar study needs to be conducted with larger samples in different study groups. Such studies could help determine the effects of sesame oil on phlebitis more clearly and strengthen the results.

## AUTHOR CONTRIBUTIONS

Muaz Gülşen wrote the protocol, performed the data collection, the statistical analyses and drafted the manuscript. Muaz Gülşen and Sevban Arslan performed the data processing, was involved in the statistic analyses and critically revised the article. Muaz Gülşen collected the data and critically revised the article. Muaz Gülşen and Sevban Arslan conducted protocol development, submission and regulatory aspects and critically revised the article. Sevban Arslan was involved in the study supervision and critically revised the manuscript. Muaz Gülşen designed and supervised the study, was involved in manuscript drafting and revision. All the authors read and approved the final manuscript.

## PATIENT CONSENT STATEMENT

The patients who accepted to participate in the study were informed about the purpose of the study and the procedures to be performed, and they signed the ‘Informed Consent Form’.

## Supporting information


**File S1.** CONSORT 2010 checklist of information to include when reporting a randomised trial.

## Data Availability

The data that support the findings of this study are available on request from the corresponding author. The data are not publicly available due to privacy or ethical restrictions. Researchers must use the consent form determined by the competent authority that conducts the ethical evaluation of the research. In this form, the identity and contact information (confidential information) of the participants must be kept confidential. Otherwise, legal sanctions are imposed on researchers. The form determined and implemented by the Ethics Committee has been added as an additional file (Turkish/English) to the "Patient Consent Form" tab.
